# Proteomic Analyses Discern the Developmental Inclusion of Albumin in Pig Enamel: A New Model for Human Enamel Hypomineralization

**DOI:** 10.3390/ijms242115577

**Published:** 2023-10-25

**Authors:** Ana Gil-Bona, Hakan Karaaslan, Baptiste Depalle, Rosalyn Sulyanto, Felicitas B. Bidlack

**Affiliations:** 1 The Forsyth Institute, 245 First Street, Cambridge, MA 02142, USA; 2 Department of Developmental Biology, Harvard School of Dental Medicine, 188 Longwood Avenue, Boston, MA 02115, USA; 3 Department of Dentistry, Boston Children’s Hospital, 300 Longwood Avenue, Boston, MA 02115, USA

**Keywords:** tooth enamel proteins, enamel proteomics, dental development, protein time stamp, fetal albumin, chalky teeth, molar hypomineralization, molar incisor hypo-mineralization, demarcated enamel opacity, porcine animal model, developmental dental defect, mineral maturation, post-eruptive enamel hardening

## Abstract

Excess albumin in enamel is a characteristic of the prevalent developmental dental defect known as chalky teeth or molar hypomineralization (MH). This study uses proteomic analyses of pig teeth to discern between developmental origin and post-eruptive contamination and to assess the similarity to hypomineralized human enamel. Here, the objective is to address the urgent need for an animal model to uncover the etiology of MH and to improve treatment. Porcine enamel is chalky and soft at eruption; yet, it hardens quickly to form a hard surface and then resembles human teeth with demarcated enamel opacities. Proteomic analyses of enamel from erupted teeth, serum, and saliva from pigs aged 4 (*n* = 3) and 8 weeks (*n* = 2) and human (*n* = 4) molars with demarcated enamel opacities show alpha-fetoprotein (AFP). AFP expression is limited to pre- and perinatal development and its presence in enamel indicates pre- or perinatal inclusion. In contrast, albumin is expressed after birth, indicating postnatal inclusion into enamel. Peptides were extracted from enamel and analyzed by nano-liquid chromatography-tandem mass spectrometry (nanoLC-MS/MS) after tryptic digestion. The mean total protein number was 337 in the enamel of all teeth with 13 different unique tryptic peptides of porcine AFP in all enamel samples but none in saliva samples. Similarities in the composition, micro-hardness, and microstructure underscore the usefulness of the porcine model to uncover the MH etiology, cellular mechanisms of albumin inclusion, and treatment for demarcated opacities.

## 1. Introduction

In healthy human enamel development, albumin is excluded from the enamel space because it hinders its hardening. This study shows that albumin is included during normal development of pig teeth that erupt with soft, chalky, and opaque enamel and quickly form a shiny and hard enamel surface after eruption. These findings resolve the long-standing question whether albumin is a post-eruptive enamel contamination. The study highlights the value of a porcine model to resolve the etiology of albumin inclusion and the possibilities of removal to harden lesions in human enamel defects.

Tooth enamel is the outer layer of the tooth crown and, at the time of eruption, is a cell-free fossil record of its own formation, tracking the apposition and mineralization of extracellular matrix and recording many aspects of our life history [1,2]. While we have a good sense of when teeth form during human ontogeny, we rely on animal models to understand the mechanisms of healthy as well as disrupted tooth formation and mineralization [3,4]. The urgency of model systems that advance mechanistic insights and effective treatment has come into focus with the astounding prevalence and unresolved etiology of a developmental dental defect reported to affect between one in six and one in eight children globally [5,6,7]. This prevalent dental defect manifests as sporadic and localized demarcated lesions of soft enamel in primary and permanent teeth.

These demarcated opacities are indicative of soft, protein rich, and hypomineralized enamel and the condition is known as molar hypomineralization (MH) or molar incisor hypomineralization (MIH). We prefer the term MH because demarcated opacities can occur in molars only. The defective enamel areas are most seen on the second primary molars, also known as two-year molars based on their time of eruption, and the first molars of the permanent dentition, that is the 6-year molars, as demarcated opaque lesions that range in color and severity from white in mild forms to cream colored, yellow, and brown [8,9,10]. Teeth with demarcated opacities are often associated with increased pain sensitivity that compromises diet and eating behavior, oral hygiene, and tolerance of dental exams [11].

The discoloration and softness of enamel within a demarcated opacity are caused by excess protein. Typically, the enamel-forming cells, ameloblasts, form an epithelial barrier that defines a tightly regulated space for the secreted enamel matrix. In healthy human enamel, the enamel matrix contains many proteins, but not serum albumin, that regulate the size, shape, and composition of enamel crystals [2]. Enzymes facilitate the removal of proteins from the enamel space, hereby allowing the growth and expansion of the crystals until only about 1% by weight of protein remains and the space is filled with tightly packed crystals [12]. It is the dense packing of crystals and the very low protein content that make enamel the hardest substance in the human body, about as hard as carbonated steel [13]. Enamel with higher protein content is more opaque and softer. The elevated protein content in demarcated opacities includes albumin that leaked into the enamel space. The intrusion of albumin into forming enamel arrests mineralization and prevents the complete hardening of enamel before eruption [14,15,16]. Albumin has this deleterious effect because it binds with high affinity to calcium phosphate crystals and prevents their further growth [17,18,19,20].

The process of enamel formation is similar in different mammals with respect to molecular machinery and mineralization and the porcine model has been widely used to study enamel formation and to extract enamel matrix proteins from forming teeth for in vitro studies [21,22,23,24,25,26]. However, the speed of enamel matrix secretion and crown formation varies greatly and, compared to humans, is at least four times faster in pigs [27,28,29].

In the fast-developing pigs, the duration of preeruptive enamel maturation and hardening is greatly reduced and pig teeth are known to erupt with softer enamel [27,28,29].

Excess protein and the high affinity of albumin for enamel crystallites diminish the efficacy of fluoride treatments and decrease the bonding of dental fillings [30,31,32,33,34,35,36]. The transfer of strategies from caries treatment has a high failure rate because the properties of hypomineralized enamel lesions are distinct from caries and require targeted treatment strategies [35,37,38,39,40].

Despite recent breakthroughs in our understanding of what demarcated opacities are and their pathophysiology [14], several key questions need to be resolved to unravel the etiology and find paths towards medical prevention of demarcated opacities. First, what causes the ameloblasts to lose their barrier function, become leaky, and allow the penetration of albumin into the enamel space? To address this, mechanistic studies on forming teeth and ameloblast physiology are required. Second, how can the excess protein be removed to allow for the expansion of enamel crystals and to achieve healthy hardness? Strategies for treatment need to be developed and tested for clinical application. Dental approaches that work well for caries treatment have limited success and a high failure rate for treating demarcated opacities [41]. This is due to the enamel breakdown in severely hypomineralized enamel, the diminished bonding of fillings, and the limited penetration of glass ionomers that are sequels of the high protein content [42,43].

In humans, primary teeth start to form in utero and tooth formation occurs inside the jaws and the erupted tooth enamel is cell-free [3,4]. The study of cellular processes during tooth formation as well as the study of new applications to treat enamel hypomineralizations require the use of model systems. An additional and major challenge for the efficient development and optimization of a targeted treatment is access to hypomineralized human teeth without restorations. Teeth from MH patients are usually extracted after repeated restorative procedures and, at that point, often lack the demarcated opacities that are the needed experimental target [34,44].

Some rodent models have been reported with promising similar characteristics to demarcated opacities [45,46]. While the murine model is genetically tractable, important drawbacks such as minute sample size, thin enamel, and different enamel structure compared to human enamel limit its usefulness to test treatment strategies and therapeutical approaches [47]. Demarcated enamel opacities and lesions with soft enamel have been produced in a sheep model with severe gastroenteritis [48]. This model has the great advantage of a big tooth size that is more similar to the size of human teeth although the thin enamel layer and the severe physiological stress causing the demarcated enamel lesions are drawbacks [49,50].

In this study, we ask whether the pig model has advantages that make it ideal for studies of molar hypomineralization and hypomineralized tooth enamel. Our objective is to examine whether pig teeth are similar enough to human teeth in terms of their size, enamel structure, and composition to study causes, possibilities for prevention, and repair of hypomineralized human enamel and chalky teeth. Practical clinical considerations and similar chemical and mechanical properties would favor the porcine model [51,52,53,54,55].

Addressing the urgent need for a suitable animal model for dental defects and, specifically, MH, herein, we tested (A) whether the protein content in pig teeth would make this model especially useful as a model to study mechanisms of albumin-related mineral poisoning [14] and (B) whether the porcine model is suitable to develop better treatment modalities since pig enamel is soft and protein-rich at the time of eruption, yet, it gains quickly in hardness after eruption [29].

## 2. Results

### 2.1. Porcine Teeth Are Similar to Human Teeth in Size and Shape

**Figure 1:**
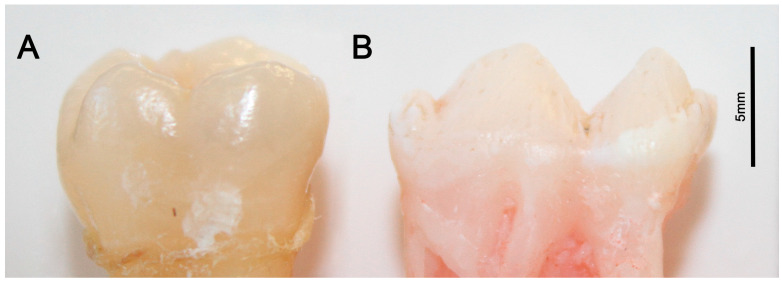
The similar size range of tooth crowns with more than 5 mm in height and about 1 cm in crown length is evident in the human molar (**A**) side by side with a pig premolar (**B**). Scale in mm.

The simple comparison between a human healthy permanent molar, the tooth type with the most common presentation of hypomineralized lesions of MH, and a porcine primary premolar shows a comparable size range (Figure 1).

### 2.2. Porcine Teeth Show Opaque Enamel and Localized Opacities Similar to Demarcated Opacities in Human Teeth

**Figure 2:**
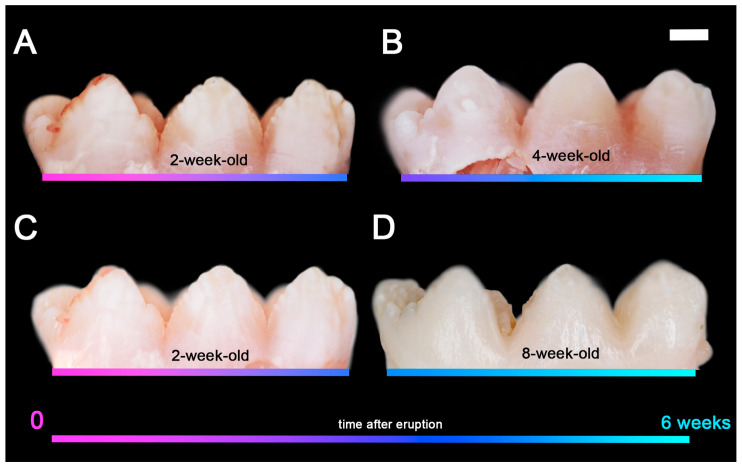
The enamel surface of pig teeth changes with time after tooth eruption from opaque and porous, seen in the fourth primary premolar of a 2-week-old pig under polarized light (**A**), and without polarization (**C**) towards a less porous and increasingly shiny surface of the same tooth type in a pig at 4 weeks of age (**B**). After an additional 4 weeks of post-eruptive changes, the tooth surface is shiny and hard (**D**). Color bars represent the time of enamel exposure after eruption. Magenta: before eruption; blue: erupted; cyan: 6 weeks post-eruption. For a description of changes in surface hardness and microstructure, see [29].

**Figure 3:**
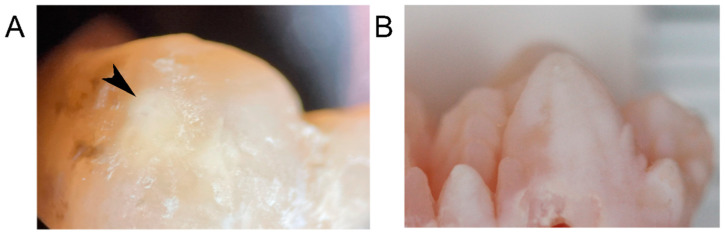
(**A**) A 6-year human molar (first molar in adult dentition) seen under polarized light macrophotography shows localized hypomineralization (black arrowhead) that is similar to demarcated opacities in the enamel of a deciduous premolar of a 4-week-old pig (**B**). Analyses of this tooth revealed the albumin content typical for human demarcated opacities.

Porcine teeth initially have an opaque surface, indicative of high porosity and associated with high protein content in enamel, which is visible in macrophotography images taken under white and polarized light (Figure 2). The tooth surface changes within weeks after eruption from opaque and porous in two-week-old animals to a tooth crown with less opaque and more shiny areas in 4-week-old pigs to a crown surface that is shiny and hard at 8 weeks after birth (Figure 2). In addition, a close-up view under polarized light shows localized opacities on the premolar tooth crown of a 4-week-old animal (Figure 3B) that are reminiscent of demarcated opacities in human teeth (Figure 3A).

### 2.3. Hardness and Lose Packing of Enamel Rods Are Similar in Pig Enamel and Human-Demarcated Opacities

**Figure 4:**
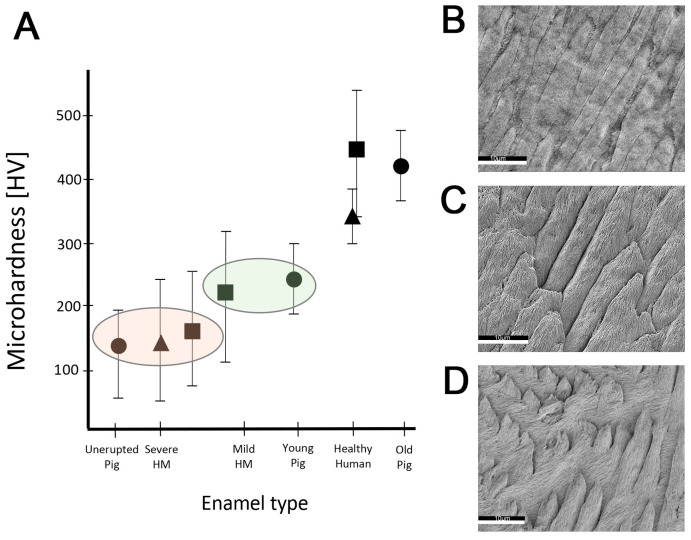
(**A**) Comparison between microhardness values reported for human demarcated opacities, healthy human enamel, and data acquired from pig teeth used in this study. The microhardness values of young pig enamel range with values for mild demarcated opacities in human teeth and are clearly below healthy human enamel. Triangles: data from Fagrell et al., 2010 [52]; Squares: data from Crombie et al., 2013 [38]; Circles: data measured on pig teeth analyzed in this study. (**B**–**D**) Scanning electron microscopy images of (**B**) healthy human enamel, (**C**) hypomineralized enamel from a demarcated opacity, (**D**) and healthy porcine enamel. Similar gaps between bundles of enamel crystallites and etching patterns are seen in (**C**,**D**) and indicate elevated acid solubility. Scale bars: 10 μm; Magnification 2300×.

Young pig enamel has an average hardness in Vickers units (HV) of 234 HV (SD = 62) with a range of 95–294 HV (*n* = 3). The microhardness values were slightly lower in the areas closer to the surface. This value is close to the hardness values reported for mild demarcated opacities in human teeth and clearly below the average values of 300 HV for healthy human enamel (Figure 4A).

Scanning electron microscopy images from healthy human enamel showed tightly packed enamel rods with minimal gaps between enamel rods (Figure 4B). In contrast, the porcine enamel appeared to be packed less densely and exhibited more space between enamel rods (Figure 4D). The etching pattern of porcine teeth was similar to that observed in human enamel within a demarcated opacity (Figure 4C). Hypomineralized human enamel showed a deeper etching with more loss of material between enamel rods and with crystallites being smaller and more corrugated compared to the healthy human enamel in the same tooth (Figure 4B,C).

### 2.4. Fetal Serum Albumin, Alpha-Fetoprotein (AFP), Is Present in Porcine Enamel and Is of Developmental Origin

The total number of proteins in porcine enamel with more than two peptides identified decreases with the age of the pig (Table 1) and varies between samples from 216 to 433, as summarized in Table 1. The AFP sequence in pigs is 610 amino acids long with 59 trypsin cleavage sites predicted by PeptideCutter (Swiss Institute of Bioinformatics) (Supplementary Sheet S1). We identified 13 different AFP peptides in all analyzed teeth by MS (Table 2).

**Figure 5:**
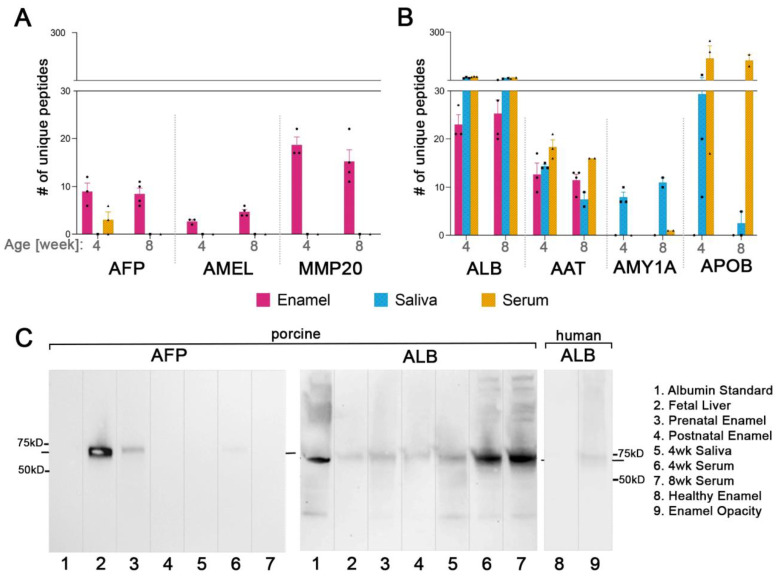
Comparison of abundance of selected proteins in enamel, saliva, and serum of 4- and 8-week-old pigs. (**A**) Number of unique peptides from AFP (alpha-fetoprotein, fetal albumin), AMEL (amelogenin), and MMP-20 (matrix metalloproteinase-20). (**B**) Number of unique peptides from ALB (serum albumin), AAT (alpha-1-antitrypsin), AMY1A (alpha-amylase), and APOB (apolipoprotein-B). (**C**) Western blot for AFP and ALB. Lanes: (1) porcine albumin standard, (2) fetal liver, (3) AFP(+) enamel that developed prenatally from 4-week-old pigs, (4) AFP(−) porcine enamel developed postnatally, (5) saliva from 4-week-old pigs showing only the adult form of albumin, (6) AFP(+) serum obtained from 4-week-old pigs, and (7) serum without AFP from 8-week-old pigs. The bands of porcine AFP and ALB are consistent with molecular weights of 68.6 for AFP and 69.7 kD for ALB.

In all samples, AFP was partially cleaved as only 13 different unique tryptic peptides were identified, covering 21.48% of the sequence (131 amino acids out of 610) (Supplementary Sheet S1). We identified 12, 6, and 9 unique peptides per tooth in the three 4-week-old pig enamel samples. In the 8-week-old pigs, the number of unique tryptic peptides of AFP varied between teeth with 10, 7, 6, and 11 tryptic peptides (Table 2). Figure 5A shows the mean number of unique AFP peptides identified per age group in comparison to amelogenin (AMEL), matrix metalloproteinase-20 (MMP-20), serum albumin (ALB), and salivary proteins. The sum of intensities of AFP peptides in 4- and 8-week-old pigs, a semi-quantitative indication of abundancies, and total identified peptides were consistent with the number of unique peptides (Supplementary Figure S1).

### 2.5. Fetal Albumin Is a Time Stamp for Natural Inclusion into Enamel Formed before Birth and Is Not Found in Enamel That Formed after Birth

Western blot analysis of the 4-week-old porcine premolars confirmed the MS-identified AFP (Figure 5C, Lane 3) with a band at around 68.6 kD which is the molecular weight of AFP reported in the literature. The absence of AFP in the postnatally developed porcine tooth further supports our findings (Figure 5C, Lane 4). Saliva and blood samples of the same 4- and 8-week-old animals used for tooth analyses were analyzed by MS to discern whether albumin in tooth enamel is of developmental origin or results from contamination in the oral cavity. We identified three and six unique AFP peptides in the serum of two of the three analyzed 4-week-old pigs while no AFP peptides were identified in the saliva or serum in the other 4-week-old or any of 8-week-old pig (Table 2).

Western blot analysis confirmed the absence of AFP in saliva samples and blood samples from the AFP(−) 4-week-old pigs (Figure 5C: Lane 5,7). We also confirmed the AFP presence in one of the AFP(+) blood samples (Figure 5C: Lane 6) and did not see any related bands at 68.6 kDa, the AFP protein size, in the serum albumin standard (Figure 5C: Lane 1) while the fetal liver lane showed a strong signal (Figure 5C: Lane 2). No cross-reactivity was seen between anti-AFP antibody and porcine serum albumin.

**Figure 6:**
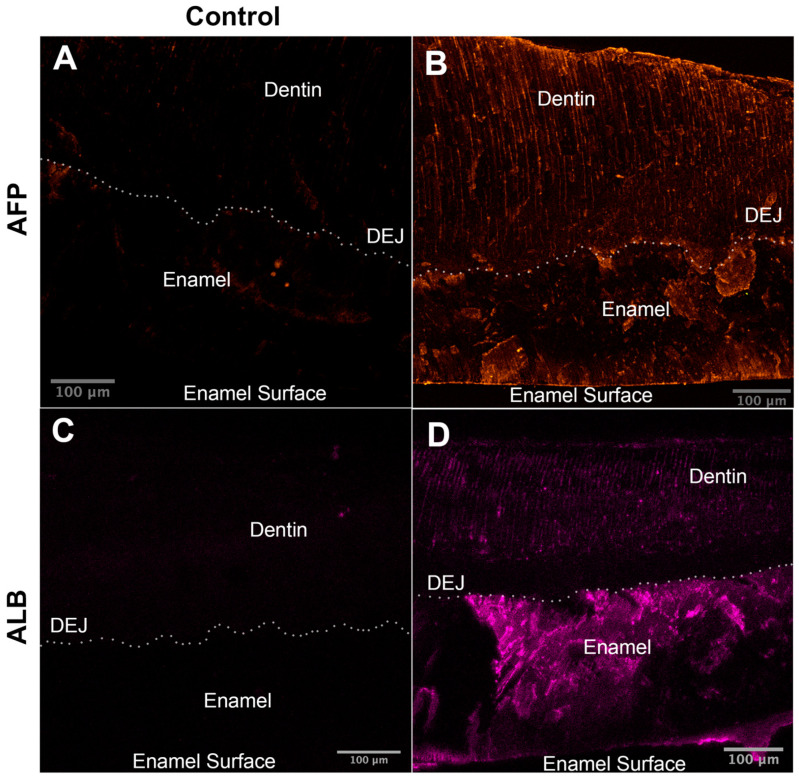
Immunofluorescence images of premolars from 8-week-old pigs labeled using anti-AFP (anti-alpha-fetoprotein, anti-fetal albumin) and anti-ALB (anti-serum albumin) antibodies. Images represent the maximum intensity projections of Z-stacks with identical image numbers and spacing. (**A**) Negative control for AFP. (**B**) Distribution of AFP is higher throughout enamel but also seen in dentin. (**C**) Negative control for ALB. (**D**) ALB is seen in enamel and dentin. Dotted lines indicate the dentin–enamel junction. E: Enamel; ES: Enamel Surface; D: Dentin.

We located ALB and AFP by immunofluorescence imaging in the enamel of 4- and 8-week-old pigs, as seen in Figure 6. No AFP or ALB signal was detected from either of the negative controls. Even distribution of AFP was observed in enamel and dentin while a decrease in ALB levels from the enamel surface to dentin was observed.

### 2.6. The Adult Form of Serum Albumin Occurs Naturally in Enamel and Is Detected in Serum, Saliva, and Enamel That Formed at Different Times

Peptides from serum albumin were identified in all enamel, saliva, and serum samples analyzed. A comparison between age groups and the type of samples shows that an average of 75, 159, and 1508 total ALB peptides were identified in enamel, saliva, and serum samples, respectively (Supplementary Figure S1). The mean number of identified unique ALB peptides was 24 in enamel, 42 in saliva, and 47 in serum samples (Figure 5B). The amount of albumin was very high in serum compared to in saliva and enamel. In all porcine samples, the number of total and unique ALB peptides identified was lower in enamel than in both saliva and serum (Figure 5A).

Western blot analysis confirmed the presence of adult albumin in the porcine enamel, saliva, and serum samples (Figure 5C: Lanes 3–7). Adult albumin was also detected in the fetal liver extract, albeit in lower quantities (Figure 5C: Lane 2). The adult form of human albumin was also detected in the demarcated opacity on human enamel while the intact enamel on the same tooth did not produce any bands (Figure 5C: Lane 8,9).

### 2.7. Common Proteins from Serum and Saliva Are Not Seen Leaking into Enamel as Contamination and Were Not Detected in Erupted Tooth Enamel

We evaluated for the presence of abundant saliva and serum proteins in enamel to discern post-eruptive exchange with exogenous proteins after enamel formation is completed. As shown in Figure 5B, alpha-amylase was detected in all saliva samples of all pigs but not in any of their enamel. Apolipoprotein B-100 was highly abundant in serum and in saliva but no signal was detected in enamel samples from any of the pigs. We identified alpha-1-antitrypsin in enamel samples; on average, 14 peptides from alpha-1-antitrypsin were identified in both 4- and 8-week-old pig enamel while 12 were identified in their saliva and 30 in their serum (Figure 5B).

### 2.8. Opaque Pig Enamel Is Not Enriched in Endogenous Enamel Proteins and Enzymes

**Figure 7:**
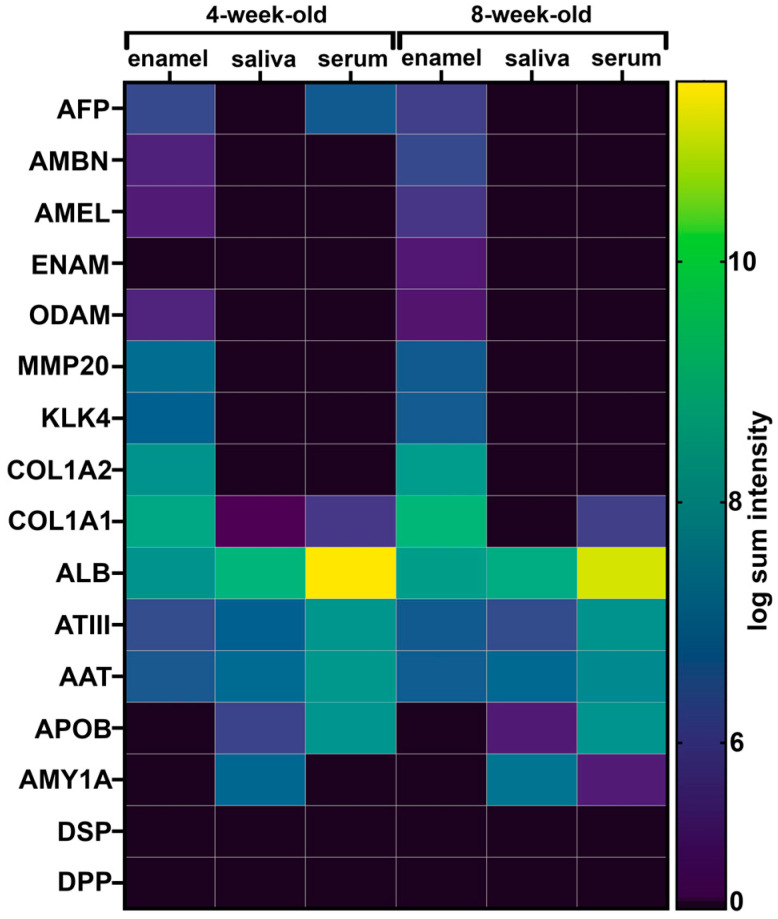
Mean sum intensities of the peptides identified for selected proteins representing tissue specificity of enamel, dentin, serum, and saliva. Color scale represents logarithmic values. DSP: Dentinsialoprotein, DPP: Dentinphosphoprotein, ODAM: Odontogenic ameloblast-associated protein, COL1A2: Collagen 1A1, COL1A2: Collagen 1A2, ATIII: Antithrombin III.

All three structural enamel matrix proteins amelogenin, ameloblastin, and enamelin and the enamel proteases MMP-20 and kallikrein-related peptidase 4 (KLK-4) were identified in porcine enamel (Figure 7). A mean number of two and five peptides of ameloblastin were identified in the 4- and 8-week-old pig teeth, respectively. Enamelin was only identified in one of the 8-week-old pig’s enamel, with four peptides. On average, three peptides from amelogenin were identified in 4-week-old pigs and six peptides were identified in 8-week-old pigs. MMP-20 was identified with an average of 20 and 18 peptides in 4- and 8-week-old pigs, respectively. KLK-4 was identified with four peptides in the enamel of both 4-week-old and 8-week-old pigs on average. None of the enamel proteins or proteases were identified in saliva or serum samples (Figure 7).

## 3. Discussion

The characteristics and pathophysiology of demarcated enamel opacities have recently been described highlighting albumin in the enamel space as the main culprit for disrupted enamel maturation with arrested crystal growth. However, animal models are urgently needed to resolve the cellular processes of etiology and mechanisms of albumin intrusion into the developing enamel that are still unresolved [20,56,57,58,59]. To address this need, we focused our analyses of pig teeth on aspects of tooth size, enamel opacity and associated surface properties, enamel microstructure, and the abundance and composition of proteins in enamel. We compared pig enamel to hypomineralized human enamel to evaluate the advantages of the porcine model for studies of causes and treatments for hypomineralized enamel (Table 3).

### 3.1. The Similarity in Size between Human and Porcine Teeth Is Important for a Useful Model System and for Testing of Treatment

The similarities between human and porcine teeth in size, shape, and enamel thickness overcome limitations of the rodent model to study the delivery and exchange of agents to harden the enamel layer.

The natural and fast surface transition from porous opaque to hard and shiny enamel in pig teeth within weeks after eruption is promising for strategies of enamel hardening. At the time of eruption, porcine teeth have enamel with an unusually low mineral density and resemble hypomineralized human enamel [26,27]. The opacity of the entire porcine tooth crown suggests that porcine enamel is a natural model for hypomineralized enamel and human enamel opacities. Additional properties underscore the advantages of the porcine model, such as the natural and fast gain in mineral density that hardens the enamel surface within two weeks after tooth eruption [29]. These porcine enamel properties, including the post-eruptive changes, meet the D3G definition of both types of demarcated opacity defects [9,15]. Our findings highlight the advantages of the porcine model and potential new insights into how the very fast pace of porcine enamel formation contributes to leakage of albumin into enamel and how enamel can harden within two weeks after eruption. 

### 3.2. The Similarity in Protein Content between Porcine and Hypomineralized Human Enamel Is Key for a Good Animal Model

We present evidence for the developmental origins of albumin in porcine enamel based on the distinction between fetal and adult forms of albumin similar to previous studies [16]. Using AFP as a temporal marker to discern between albumin inclusion during tooth development and later contamination, we show the similarity between opaque porcine enamel and human hypomineralized enamel from demarcated opacities. Additionally, we show differences in protein composition in the aging teeth consistent with our previous analyses of porcine enamel and changes in albumin distribution characterized in demarcated opacities in human enamel [2,15,60].

The similarity in lose packing and low hardness of pig enamel compared to hypomineralized human enamel highlights the advantage of the porcine model. The microhardness of porcine enamel is lower than healthy human enamel and similar to that of mild demarcated opacities [52,61]. A comparison of enamel microstructure after acid etching shows gaps between rods of enamel crystals in porcine enamel similar to human hypomineralized enamel in contrast to the densely packed healthy human enamel rods. This etching pattern was described previously and was associated with the increased solubility of inter-rod higher enamel possibly caused by elevated protein content [62,63]. The higher abundance of organic material in porcine and hypomineralized human enamel is consistent with increased acid solubility, higher porosity between rods, and more corrugated crystallites within the rods (Figure 4C,D).

**Table 3:** Comparison of tooth properties in different animal models and their suitability as models for the study of human enamel defects, especially demarcated opacities in molar hypomineralization.

	Human	Pig	Mouse	Zebrafish
**Crown Size** **(height)**	5–10 mm [4,64]	5–12 mm [65,66]	Molars: 1–2 mm; Incisor: >1 cm [67]	Continued replacement: <100 microns [68]
**Duration of** **crown mineralization before eruption**	Primary: 10 to 36 months (incisors to molars) Permanent: 30–78 months (first molar to canines) [69]	Primary: 3–4 months Permanent: 4–12 months [70]	2.5 weeks [67] Only one set of teeth	2 weeks [68] Continuous tooth replacement
**Enamel Mineral** **Density** **[g/cc]**	Primary: 1.6–2.2 [71] Permanent: 2.1–3.1 [72];	Primary: 1.6–2.0 [29] Permanent: 2.8–2.9 [28]	Incisor tip: 3.0 [73]	No enamel
**Hardness** **[GPa]**	Primary: 3.0 [62] Permanent: 3.6–4.1 Hypomineralized: 1.4–2.1 [52,61]	Primary: 3.4 Permanent: 3.9–4.1 [29]	Incisors: 3–4 [74]	No enamel
**Micro-structure**	Decussating Rod/Interrod keyhole pattern [75]	Rods/interrod sheets [55]	Rod/interrod Sheath [76]	No enamel
**Posteruptive** **maturation**	Little [77,78,79]	Extensive [29]	Little [47]	No enamel
**Protein matrix** **composition in** **healthy,** **erupted teeth**	Healthy: Enamel matrix proteins, no albumin [2] Albumin in enamel opacities [15]	Similar to hypomineralized human enamel with albumin in erupted enamel [60]	Similar to healthy mature human enamel, no albumin [2]	No Enamel
**Major limitation** **for use in studies**	Limited access to untreated hypomineralized samples	Cost for large animal model in controlled facility	Small sample size	Enameloid, not enamel, small size
**Major advantage**	Ideal sample for studies of etiology and treatment in humans	Similar tooth properties; ease for proteomic, molecular pathway studies; genetic tractability;	Ease of genetic modifications and tractability; molecular pathways.	Ease of genetic, molecular pathways and toxicology studies [80]

Fetal serum albumin in porcine enamel indicates developmental albumin inclusion comparable to human enamel demarcated opacities. Albumin in porcine enamel and diminished hardness compared to healthy human enamel were described previously [19,27]. Our findings are in line and support the description of the high albumin content in pig enamel by Kirkham and Robinson (1988) who also pointed out the fast formation times of pig teeth as a cause for the incomplete maturation of enamel [27]. It has been a long-standing question as to whether albumin in enamel is of developmental origin or a contaminant introduced during sample harvesting. We show that teeth that formed before birth but were harvested well after birth contain AFP must, therefore, be included during tooth development.

To discern between potential contaminations and exchange processes that naturally occur during post-eruptive enamel maturation, we examined the presence of salivary proteins in enamel (Figure 5B) [81]. Alpha amylase is an abundant protein in saliva that has an affinity to hydroxyapatite similar to albumin [82,83]. Fragments of this protein were detected in human-demarcated opacities with a broken surface layer but were not seen in the ones with an intact surface, implying their penetration into the porous enamel [15]. We found alpha-amylase in the saliva of the pigs but it was not detected in their enamel. Apolipoprotein-B 100 has a lower affinity to hydroxyapatite than albumin [84] and is also one of the most abundant proteins of saliva and serum. Again, we did not detect this protein in the enamel but in the serum and saliva of the same animal. These findings or porcine enamel are consistent with human-demarcated opacities with a highly mineralized enamel surface as described by Perez et al. (2020) [15].

The total number of proteins identified in porcine enamel and the number of most common proteins decreases with tooth age. This is consistent with both our previous findings of porcine enamel and reports of post-eruptive enamel maturation [60]. In developing enamel, the enzymatic cleavage of enamel matrix proteins is critical to allow for the removal of proteins from the developing enamel through endocytosis to achieve the growth of crystallites and full enamel hardness [85,86,87]. The relatively low abundance of amelogenin, ameloblastin, and enamelin seen in porcine teeth is consistent with the literature and the expected abundance of these proteins in erupted enamel [60]. The high protein content seen in porcine enamel results from an elevated abundance of proteins that are not enamel specific, particularly albumin forms [2,31]. According to our results, another non-enamel protein contributing to the elevated organic content in porcine enamel is collagen. It has been reported in healthy enamel and in demarcated opacities previously [17,88]. The lack of dentin-specific proteins DSP and DPP in any of our enamel samples suggests that we were able to collect enamel precisely and without dentin contamination (Figure 7).

### 3.3. As in Demarcated Opacities, Pig Enamel Contains Inhibitors of KLK4, the Key Enzyme for Enamel Maturation

Supported by Western blot analyses, we found antithrombin-III and alpha-1-antitrypsin in enamel samples (Figure 7). These two serine proteinase inhibitors have been reported in yellow and brown demarcated opacities [17,89]. Both are inhibitors of kallikrein-related peptidase 4 (Klk4), which is essential for the removal of enamel matrix proteins and proper enamel hardening. In contrast to these findings, Mangum et al. did not report these proteins in human demarcated opacities that have a surface layer of highly mineralized and intact enamel [18].

### 3.4. Fetal and Adult Forms of Albumin Contribute to the High Protein Content of Porcine Enamel Based on Proteomics, Immunoblotting, and Immunofluorescence

**Figure 8:**
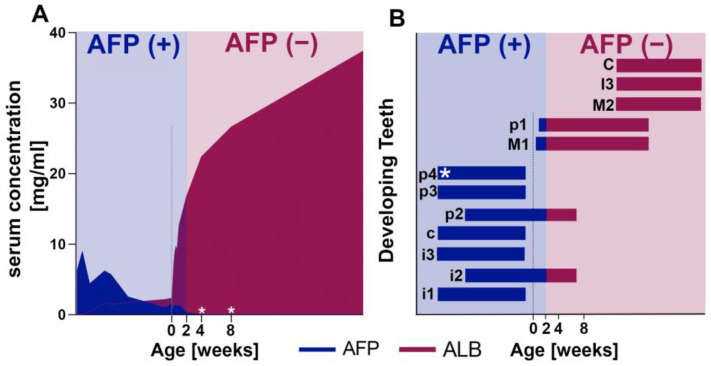
(**A**) Serum AFP and ALB concentrations of domestic pigs during gestation and after birth compiled from Cavanagh et al., 1982, and Martin et al., 2005 [90,91]. The AFP concentration decreases rapidly after birth and becomes undetectable before the pig reaches four weeks of age. The adult isoform ALB increases rapidly within days postnatally and becomes the predominant isoform in the bloodstream shortly after birth. Blue: the AFP(+) period; Red: AFP(−) period. (**B**) Pre-eruptive development of the porcine dentition; color-coded blue for development during AFP(+) and red for AFP(−) according to the presence of AFP during tooth development. Time values are approximated from the literature. Asterisks indicate the teeth used in this study. Lowercase: deciduous; uppercase: permanent; i: incisor; c: canine; p: premolar; M: molar.

In pigs, AFP expression decreases rapidly to levels below detection by four weeks after birth (Figure 8A) [90,91]. Therefore, the presence of AFP peptides in all porcine enamel samples in this study indicates an endogenous developmental origin and AFP incorporation into the developing enamel during pre- and perinatal tooth formation (Figure 8B). These findings are further supported by the detection of only the adult albumin form in teeth that started their formation postnatally.

Proteomic analyses of human enamel identified that a total of 58 proteins in both the healthy human enamel and the demarcated opacities and peptides from serum albumin were in all of the samples, although with higher abundance in the demarcated opacities (Supplementary Figure S2). The development of primary teeth coincides with AFP expression, yet, we did not detect human AFP in any of the human demarcated opacities. This might be due to the degradation of AFP within human enamel lesions as described by others [15].

### 3.5. The Porcine Model Is Uniquely Suitable to Elucidate the Etiology and Improve Treatment Protocols for MH

The similarities in dental morphology and size correspond to the similarities of an omnivorous diet in humans and pigs. Our study focused on domestic pigs that are bred for fast growth and develop their teeth faster than wild boar, which still have faster dental development than humans [65]. Pig teeth also offer, based on their size, unique possibilities to analyze the mineralizing matrix through proteomic analyses [60]. This is relevant to characterize the protein composition and degradation before and after eruption that occurs naturally or reveals the pathophysiology of a defect or changes as the result of treatment [15]. Due to the small tooth size, proteomic analyses are still challenging in murine enamel without the inclusion of dentin [92]. 

There are several limitations of this animal model. For example, proteomic analyses would benefit from additional reviewed entries in the UniProtKB database for *Sus scrofa*. While it has most of the porcine proteome identified, containing 46,219 protein entries, only 1438 have been listed as reviewed [93,94]. Some enamel proteins, such as amelogenin isoforms, are most likely not identified in the database. Although we routinely use an internal reference sample of porcine enamel, another limitation is that the proteomics analyses of porcine and human samples were run without technical replicates. We did verify the mass spectrometry results for the proteins of interest by Western blot and immunofluorescence.

## 4. Materials and Methods

All experimental procedures involving the use of animals followed the Institutional Animal Care and Use Committee (IACUC) regulations of the Tufts University Cummings School of Veterinary Medicine and The Forsyth Institute. Four-week-old piglets were still nursing at collection time. Older 8-week-old pigs were fed with a premix of 72% corn, 25% soybean, and 3% vitamin/mineral. Human specimens were collected from patients at Boston Children’s Hospital via an Institutional Review Board-approved protocol (IRB-P00039904). The number of animals and the number of samples collected from the animals were selected as triplicates. Based on our experience with tooth enamel, we found that n = 3 is sufficient to detect enamel differences. All samples were harvested within the same morning time window to minimize the effect of diurnal cycles and feeding schedules affecting saliva and blood composition in these animals raised under controlled conditions.

### 4.1. Saliva Samples

Stimulated whole saliva samples were collected from pigs aged 4 weeks (*n* = 3) and 8 weeks (*n* = 2) before the animals were sacrificed. Following our previous attempts using the rope technique [95], a saliva collection device was used (SuperSAL601, Oasis Diagnostic Corporation, Vancouver, WA, USA) and approximately 1 mL of saliva was obtained from each pig. Samples were immediately transferred onto dry ice upon collection and stored at −80 °C until being processed using a modified version of the method described elsewhere [96].

Saliva samples were centrifuged at 14,000× *g* for 10 min at 4 °C, pellets containing traces of food were discarded, and the supernatant was concentrated using a 10,000 MW filter unit (Vivaspin 500, Vivaproducts, Littleton, MA, USA) at 12,000× *g*. The retentate was washed twice in extraction buffer (8 M urea, 50 mM Tris pH 8.0, 10 mM DTT) and the proteins were resuspended in 50 mM iodoacetamide (IAA), 50 mM ammonium bicarbonate (ABC) buffer, incubated for 15 min, and centrifuged at 12,000× *g* for 15 min. The retentate was washed twice with 50 mM of ABC buffer. The concentrated proteins were resuspended in 50 mM of ABC buffer and stored at −20 °C until gel electrophoresis.

### 4.2. Blood Samples

Blood samples from 4-week-old (*n* = 3) and 8-week-old (*n* = 2) pigs were harvested immediately after intravenous injection of phenytoin pentobarbital sodium (Euthosol, Virbac, Carros, France). To reduce the blood contamination during tooth extraction and collect blood samples, the bilateral jugular veins and the carotid arteries were cut immediately after sacrifice and the flow was collected without using any anticoagulants. Following collection, samples were left to coagulate at room temperature (RT) and then centrifuged at 2000× *g* at 4 °C for 15 min. Serum was collected and a modified version of the methanol/chloroform precipitation protocol [97] was used to precipitate and clean the samples. The concentrated proteins were then resuspended in 50 mM of ABC buffer and stored at −20 °C until gel electrophoresis.

### 4.3. Fetal Liver Tissue Collection

The liver of a full-term still-born domestic pig was collected from the same facility to use as a positive control for immunoblotting AFP [98]. The specimen was stored at 4 °C until collection and the left liver lobe was dissected within 8 h of death. Then, 160 μL of lysis buffer (10 mM Tris, 100 mM NaCl, 2 mM EDTA, 0.5% CHAPS) with protease inhibitor cocktail (cOmplete mini, Roche Diagnostics, Indianapolis, IN, USA) was added to ~0.5 mL of the dissected fetal liver. The mixture was homogenized using a tissue grinder for 2 min and then kept on ice for 10 min. This step was repeated 3-times and the mixture was centrifuged at 14,000× *g* for 10 min at 4 °C. The supernatant was collected and stored at −20 °C until Western blot analysis.

### 4.4. Tooth Collection, Enamel Sampling, and Protein Extraction

Immediately after the sacrifice, the jugular vein and the carotid artery were cut bilaterally to bleed out the animal. This procedure avoided contamination of intra-oral bleeding during harvesting of mandibles and maxillae, which were instantly transferred onto dry ice for transport, then stored at −80 °C until further processing. We carefully extracted the deciduous premolars from animals at the age of 4 weeks (*n* = 3 animals, 1 tooth/animal) and 8 weeks (*n* = 2 animals, 2 teeth/animal), avoiding extracellular fluid contamination. The erupted part of each tooth crown was washed with 50 mL of phosphate-buffered saline (PBS) using a toothbrush to remove pellicles and plaque. The unerupted enamel and root surface were covered while the erupted enamel was carefully collected using tungsten carbide burs and a low-speed drill to minimize sample heating. Disposable items such as burs, weighing dishes, and toothbrushes were used individually for each tooth; surfaces and non-disposable items were cleaned with 70% ethanol between each sample. Approximately three-quarters of the outer enamel layer was removed to avoid any contamination with dentin. The collected enamel powder from each tooth was weighed and placed in a 1.5 mL tube. 

We used human teeth (*n* = 4); each tooth was extracted from a different subject diagnosed with MH and receiving dental treatment at Boston Children’s Hospital. Teeth were extracted with forceps and immediately stored at −20 °C until analysis. From any given tooth, we analyzed both the demarcated opacities and intact enamel using the unaffected enamel as a negative control.

Protein extraction was performed according to protocols from the literature and previous studies conducted by our group [60,99]. Briefly, the enamel powder was dissolved in 12% trichloroacetic acid (Thermo Fisher Scientific, Waltham, MA, USA) under agitation at 500 RPM for 48 h at 4 °C and then centrifuged at 4 °C for 45 min (2500× *g*). The acid volume was adjusted to the ratio of 1 mL of acid per 10 mg of enamel. Pellets were washed twice with cold acetone (−20 °C) and centrifuged at 13,000× *g* for 10 min. Supernatants were discarded and the protein pellet was dried at RT. Proteins were resuspended in 8 M urea followed by 50 mM ABC buffer solution and stored at −20 °C until gel electrophoresis. 

### 4.5. Protein Digestion and Sequence Analysis by LC-MS/MS

All samples were subjected to gel electrophoresis, prior to mass spectrometry (MS) analysis, using 10% Mini-Protean TGX Precast protein gels (Bio-Rad Laboratories, Hercules, CA, USA). Serum, saliva, and fetal liver tissue samples were quantified using the Micro BCA Protein Assay Kit (Thermo Fisher Scientific, Waltham, MA, USA) and loading volumes were determined according to the protein concentration. For gel loading, 10 mg of enamel equivalent enamel extract and 80 µg of protein-containing amounts of saliva and serum extracts were used. The gels were run for 10 min, subjected to Coomassie staining and de-staining protocols (Gelcode Blue Safe Protein Stair, Thermo Fisher Scientific, Waltham, MA, USA), and, finally, the whole lanes from the gel were cut by sterile and apyrogenic surgical blades on sterile surfaces into approximately 1 mm^3^ pieces. Subsequently, samples were stored at 4 °C in high-performance liquid chromatography (HPLC)-grade water using individual 1.5 mL tubes until analysis. 

The gel pieces were subjected to in-gel trypsin digestion [100] at the Taplin Mass Spectrometry Facility, Cell Biology Department, Harvard Medical School. The peptides were then subjected to electrospray ionization and were entered into an LTQ Orbitrap Velos Pro ion-trap mass spectrometer (Thermo Fisher Scientific, Waltham, MA, USA). Peptides were detected, isolated, and fragmented to produce a tandem mass spectrum of specific fragment ions for each peptide. Peptide sequences and the protein identities were determined by matching the UniProtKB database for *Sus scrofa* with the fragmentation pattern acquired by Sequest software (v.28) (Thermo Fisher Scientific, Waltham, MA, USA) [101]. The data were filtered to a 1–2% peptide false discovery rate. Proteins identified with at least two peptides were used for the analysis; any deviation from this HUPO standard is specified. The ExPASy PeptideCutter tool [102] was used to predict potential cleavage sites for trypsin in the protein alpha-fetoprotein (AFP—UniProtKB—Q8MJ76 FETA_PIG). Unique peptide numbers indicate the number of peptides that exist only in the given protein considered while the total peptide numbers indicate all the peptides identified for the given protein. Semi quantitative abundancy values are based on the sum of peak intensities of the identified peptides. Statistical analysis and data visualization were performed using Prism 9 (Graphpad Software Inc., San Diego, CA, USA). The gene ontology tool was used for enrichment analysis [103].

### 4.6. Western Blot

Western blot analysis was performed for porcine ALB and AFP in two separate gels using 10 µg of protein loaded from saliva and serum samples, fetal liver extract, and stock porcine serum albumin (A4414, Sigma-Aldrich, St. Louis, MO, USA). Six mg of a 4-week-old enamel sample (prenatal enamel) and the same amount of enamel from a postnatally developed tooth were also loaded in 4–20% Mini-Protean TGX gels. We used fetal liver extract as a positive control for AFP and porcine serum albumin as a positive control for ALB. The porcine serum albumin standard solution was also used to check the cross-reactivity of the anti-AFP antibody with ALB.

Additional analyses were performed on a human maxillary permanent first molar with white demarcated opacities and an intact surface. Six mg of enamel was collected within the opacity and from healthy areas of the crown as described above under Section 2.4 for the porcine teeth and included in the Western blot analysis, using a third gel. After gel electrophoresis was completed, proteins were transferred into membranes using the Trans-Blot Turbo Transfer System (Bio-Rad Laboratories, Hercules, CA, USA) and blocked using 5% dry milk in PBST (PBS, 0.1% Tween 20, 0.1% Sodium azide) for 1 h at RT and then incubated at 4 °C overnight with primary antibody solutions (mouse monoclonal anti-α-fetoprotein antibody (SAB4200746 Sigma-Aldrich, St. Louis, MO, USA), rabbit anti-pig albumin antibody (ab79960 abcam, Cambridge, UK), and mouse anti-human albumin antibody (A6684 Sigma-Aldrich, St. Louis, MO, USA)) at 1:500, washed with PBST, and incubated for 1 h at RT with the secondary antibody solutions (goat anti-mouse IgG HRP (#62-6520 Invitrogen, Waltham, MA, USA) and goat anti-rabbit IgG HRP (#32460 Invitrogen, Waltham, MA, USA)) at 1:10,000. Membranes were developed using a chemiluminescent HRP substrate (SuperSignal™ West Pico PLUS, Thermo Fisher Scientific, Waltham, MA, USA) and chemiluminescence was imaged using the G:BOX EF imaging system (Syngene, Frederick, MD, USA).

### 4.7. Immunofluorescence Imaging

Porcine teeth contralateral to samples used for proteomics analysis (*n* = 3) were fractured along the sagittal plane to localize AFP and ALB throughout the enamel layer. After fracture, the enamel chips were fixed using 10% zinc formalin solution, incubated with 5 mg/mL sodium borohydride to reduce free aldehydes and autofluorescence, and blocked using 5% dry milk in PBS for 1 h [104]. Samples were incubated with primary antibodies (mouse monoclonal anti-α-fetoprotein antibody SAB4200746 and goat anti-pig albumin (ab112980 Abcam, Cambridge, UK), 1:1000 dilution in blocking buffer) at 4 °C overnight, washed with PBS, and incubated with the secondary antibodies for 4 h at RT (goat anti-mouse IgG Alexa Fluor 546 (A11030 Invitrogen, Waltham, MA, USA) and donkey anti-goat IgG Alexa Fluor Plus 647 (A32849 Invitrogen, Waltham, MA, USA), 1:1000 dilution in blocking buffer). Negative controls were only subjected to secondary antibodies. Samples were imaged on a Zeiss LSM 880 Confocal Microscope (Carl Zeiss AG, Oberkochen, Germany) using 633 nm and 561 nm laser excitation and image Z-stacks were analyzed with FIJI Imaging Software (v1.53) [105].

### 4.8. Microhardness Measurements and SEM Imaging

Vickers microhardness data were collected on premolars (*n* = 3) from 4-week-old pigs. The teeth were embedded in resin (Epotek, Epoxy Technology Inc., Billerica, MA, USA), sectioned in the buccolingual plane, and both section surfaces of each tooth were polished to 0.1 µm grit size. Microhardness was measured on one polished section surface in lateral enamel with 5 s of 25 g load on a LECO M400 microhardness tester (St. Joseph, MI, USA). Indents were placed in 50 µm intervals on a transect from the enamel surface to the dentin–enamel junction (DEJ).

The other polished section surface was used for scanning electron microscopy (SEM) analyses. A human primary molar tooth with a mild demarcated opacity was included in the SEM analyses and equally prepared, i.e., resin embedded, sectioned buccolingually through the demarcated opacity, and polished to 0.1 µm grit size. The polished surfaces were etched using 0.1 M phosphoric acid for 10 s, platinum–palladium coated, and imaged at 2 kV and ×2300 magnification using a JEOL 7900F SEM (JEOL USA, Peabody, MA, USA).

### 4.9. Macrophotography

Cross-polarized macrophotographs of dry enamel were taken using a cross-polarizing filter, a ring flash, and a 65mm macro lens on a digital camera (Canon USA Inc., Huntington, NY, USA). Porcine enamel and a human mild demarcated opacity, with an intact surface, were imaged using the technique described in Robertson and Toumba (1999) [106].

## 5. Conclusions

The similarity between porcine teeth and human chalky teeth in size, enamel composition, microhardness, and microstructure highlights the potential of a porcine model to advance our understanding of dental processes involved in MH. The developmental origin of fetal serum albumin in normally formed pig tooth enamel offers opportunities to further explore the cellular mechanisms of albumin inclusion and to develop dental treatment strategies for demarcated opacities. Pig teeth erupt with opaque enamel and a soft and porous tooth surface that mineralizes rapidly after eruption into a shiny and hard tooth surface. This transformation and associated changes in enamel properties make pig teeth useful for comparison with both soft and porous as well as shiny and hard demarcated opacities in human teeth. Expected structural enamel matrix proteins and proteases were detected in all enamel samples. The presence of the fetal and adult forms of serum albumin in enamel and the absence of some common salivary proteins suggest that AFP peptides and, possibly, the adult form of albumin are incorporated and retained in developing porcine enamel. Taken together, our findings endorse the porcine model for further studies to elucidate the etiology and improve treatment protocols for MH.

## Figures and Tables

**Table 1:** Total identified proteins in 4- and 8-week-old porcine enamel and common proteins among different aged pigs.

Age	Sample	Total Proteins Identified (>2 Peptides)	Common Proteins per Age (>2 Peptides)
Enamel of 4-week-old pigs	i	342	203
	ii	237	
	iii	433	
Enamel of 8-week-old pigs	I	217	109
	II	216	
	III	409	
	IV	184	

**Table 2:** Distribution of AFP peptides (gene symbol: AFP) in all individual enamel, serum, and saliva samples from both age groups. AFP peptides were identified in all enamel samples. Gray cells: absence of the peptide. Green cells: peptide identified in the sample.

Peptides of AFP	Match to AFP Seq at Peptide No	Enamel							Serum					Saliva				
		4 Week			8 Week				4 Week			8 Week		4 Week			8 Week	
		i	ii	iii	I	II	III	IV	i	ii	iii	I	II	i	ii	iii	I	II
K.DVLTVIEK.S	69																	
R.RHPFLYAPTILSLAAQYDK.I	169																	
R.HPFLYAPTILSLAAQYDK.I	170																	
R.DFNQLSSR.E	339																	
R.FTYEYSR.R	355																	
K.LAVPVILR.V	366																	
K.GYQELLEK.C	377																	
K.YIQESQALAK.R	405																	
K.LGEYYLQNAFLVAYTK.K	423																	
K.KAPQLTPPELMALTR.K	439																	
K.KAPQLTPPELM*ALTR.K	439*																	
K.APQLTPPELMALTR.K	440																	
K.QQFLINLVK.Q	551																	
K.QKPQITEEQLEAVIADFSGLLEK.C	560																	

* Amino acid sequences.

## Data Availability

The mass spectrometry proteomics data were deposited to the ProteomeXchange Consortium via the PRIDE partner repository with the dataset identifier PXD030329.

## Supplementary Materials

The following supporting information can be downloaded at https://www.mdpi.com/article/10.3390/ijms242115577/s1.
