# Supplementary material for: Proteomic Analyses Discern the Developmental Inclusion of Albumin in Pig Enamel: A New Model for Human Enamel Hypomineralization
Source: Int J Mol Sci. 2023 Oct 25;24(21):15577. doi: 10.3390/ijms242115577 (PMC10650821; doi:10.3390/ijms242115577)
Supplement: Supplementary file 1 [file ijms-24-15577-s001.zip › Supplementary material/Figure legend for Supplementary files.docx]

Supplementary Figure 1. **(A)** Number of total peptides from AFP, AMEL and MMP20. **(B)** Number of total peptides from ALB, alpha-1-antitrypsin, alpha-amylase and apolipoprotein-B. **(C)** Sum of intensities of the peptides from AFP, AMEL and MMP20. **(D)** Sum of intensities of the peptides from ALB, alpha-1-antitrypsin, alpha-amylase and apolipoprotein-B. Filled circles, squares, and triangles: individual measurement values.

Supplementary Figure 2. Number of unique peptides from ALB, AMEL and alpha-1-antitrypsin in enamel samples. Porcine enamel and human hypomineralized enamel lesions show similarities in elevated albumin presence, amelogenin and alpha-1-antitrypsin, but differ in the number of unique peptides identified for the key enzyme MMP20.

Supplementary Sheet 1. Alpha-fetoprotein analyzed using the Expasy Peptide cutter, for tryptic peptides.
