# Supplementary material for: Proteomic Analyses Discern the Developmental Inclusion of Albumin in Pig Enamel: A New Model for Human Enamel Hypomineralization
Source: Int J Mol Sci. 2023 Oct 25;24(21):15577. doi: 10.3390/ijms242115577 (PMC10650821; doi:10.3390/ijms242115577)
Supplement: Supplementary file 1 [file ijms-24-15577-s001.zip › Supplementary material/supplementary_sheet_1.pdf]

PeptideCutter

Home | Contact

PeptideCutter

You have selected the protein FETA_PIG (Q8MJ76) from UniProtKB/Swiss-Prot :

Alpha-fetoprotein precursor (Alpha-1-fetoprotein) (Alpha-fetoglobulin)

The sequence to investigate:

```
        10         20         30         40         50         60
MKWVVSIFLI VLLNFTESRT MHENAYGIAS ILDSSQCSAE MNLVDLATIF FAQFVQEATY

        70         80         90        100        110        120
KEVNQMVKDV LTVIEKSTGS EQPAGCLENQ VSVFLEEICH EEEIPEKYGL SHCCSQSGEE

       130        140        150        160        170        180
RHNCFLARKK AAPASIPPFQ VPEPVTSCKA YEENRELFMT RYIYEIARRH PFLYAPTILS

       190        200        210        220        230        240
LAAQYDKIIP PCCKAENAVE CFQTKAASIT KELRESSLLN QHMCTVMRQF GARTFRAITV

       250        260        270        280        290        300
TKLSQKFPKA NFTEIQKLVL DVAHIHEECC RGNVLECLQD AERVVSYVCS QQDTLSSKIA

       310        320        330        340        350        360
ECCKLPTTLE LGQCIIHAEN DDKPEGLSPN LNRFLGERDF NQLSSREKDL SMARFTYEYS

       370        380        390        400        410        420
RRHPKLAVPV ILRVAKGYQE LLEKCSQSEN PLECQDKGEE ELEKYIQESQ ALAKRSCGLF

       430        440        450        460        470        480
QKLGEYYLQN AFLVAYTKKA PQLTPPELMA LTRKMATTGA ACCHLSEDRQ LACGEGAADL

       490        500        510        520        530        540
IIGQLCIRHE EMPINPGVGQ CCTSSYANRR PCFSSLVLDE TYVPPPFSDD KFIFHKDLCQ

       550        560        570        580        590        600
AQGVALQTMK QQFLINLVKQ KPQITEEQLE AVIADFSGLL EKCCQGQEQE VCFAEEGPAL

       610
ISKTRASLGV
```

The sequence is 610 amino acids long.

Available enzymes

The enzyme(s) that you have chosen:

- Trypsin

You have chosen to display all possible cleaving enzymes.

These enzymes cleave the sequence:

Name of enzyme	No. of cleavages	Positions of cleavage sites
Trypsin	59	2 19 61 68 76 107 121 128 129 130 149 155 161 168 187 194 205 211 214 228 233 236 242 246 249 257 271 283 298 304 333 338 346 348 354 361 365 373 376 384 397 404 414 415 422 438 439 453 454 469 488 509 531 536 550 559 582 603 605

These are the cleavage sites of the chosen enzymes and chemicals mapped onto the entered protein sequence:

- You have chosen a block size of **60** for the map.
- Please note that the cleavage occurs at the **right side** (C-terminal direction) of the marked amino acid.
- You have the possibility to display the results of a single enzyme by **mouseclicking** on the respective enzyme name in the map.

```
     Tryps            Tryps

         |                |
        MKWVVSIFLIVLLNFTESRTMHENAYGIASILDSSQCSAEMNLVDLATIFFAQFVQEATY
    1   ---------+---------+---------+---------+---------+---------+   60


    Tryps  Tryps   Tryps                          Tryps

        |      |       |                              |
        KEVNQMVKDVLTVIEKSTGSEQPAGCLENQVSVFLEEICHEEEIPEKYGLSHCCSQSGEE
   61   ---------+---------+---------+---------+---------+---------+   120


             Tryps
            Tryps|
    Tryps  Tryps||              Tryps Tryps Tryps  Tryps

        |      |||                  |     |     |      |
        RHNCFLARKKAAPASIPPFQVPEPVTSCKAYEENRELFMTRYIYEIARRHPFLYAPTILS
  121   ---------+---------+---------+---------+---------+---------+   180


                                                           Tryps
                                     Tryps              Tryps  |
          Tryps  Tryps      Tryps Tryps  |         Tryps    |  |

             |      |          |      |  |             |   |  |
        LAAQYDKIIPPCCKAENAVECFQTKAASITKELRESSLLNQHMCTVMRQFGARTFRAITV
  181   ---------+---------+---------+---------+---------+---------+   240


            Tryps
         Tryps  |
     Tryps   |  |   Tryps         Tryps       Tryps          Tryps

         |   |  |       |             |           |              |
        TKLSQKFPKANFTEIQKLVLDVAHIHEECCRGNVLECLQDAERVVSYVCSQQDTLSSKIA
  241   ---------+---------+---------+---------+---------+---------+   300


                                         Tryps     Tryps
       Tryps                        Tryps    |   Tryps | Tryps

           |                            |    |       | |     |
        ECCKLPTTLELGQCIIHAENDDKPEGLSPNLNRFLGERDFNQLSSREKDLSMARFTYEYS
  301   ---------+---------+---------+---------+---------+---------+   360


        Tryps      Tryps                                  Tryps
    Tryps   |   Tryps  |   Tryps        Tryps  Tryps     Tryps|

        |   |       |  |       |            |      |         ||
        RRHPKLAVPVILRVAKGYQELLEKCSQSENPLECQDKGEEELEKYIQESQALAKRSCGL
```

```
361    ---------+---------+---------+---------+---------+---------+    420



                   Tryps          Tryps
  Tryps           Tryps|         Tryps|          Tryps

     |                ||             ||              |
   QKLGEYYLQNAFLVAYTKKAPQLTPPELMALTRKMATTGAACCHLSEDRQLACGEGAADL
421    ---------+---------+---------+---------+---------+---------+    480



                                                       Tryps
        Tryps                Tryps                Tryps     |

           |                     |                     |    |
   IIGQLCIRHEEMPINPGVGQCCTSSYANRRPCFSSLVLDETYVPPPFSDDKFIFHKDLCQ
481    ---------+---------+---------+---------+---------+---------+    540



          Tryps    Tryps                   Tryps

             |        |                       |
   AQGVALQTMKQQFLINLVKQKPQITEEQLEAVIADFSGLLEKCCQGQEQEVCFAEEGPAL
541    ---------+---------+---------+---------+---------+---------+    600



     Tryps
   Tryps |

      | |
   ISKTRASLGV
601    ---------+    610
```

*Peptides Identified in Enamel from 4-Week-Old Pig

*Peptides Identified in Enamel from 8-Week-Old Pig

*Peptides Identified in Enamel from 4 & 8-Week-Old Pigs

*Peptides Identified in Serum from 4-Week-Old Pig

Expasy is operated by the SIB Swiss Institute of Bioinformatics | Terms of Use

Back to the top
