# Supplementary figures and images for: Proteomic Analyses Discern the Developmental Inclusion of Albumin in Pig Enamel: A New Model for Human Enamel Hypomineralization
Source: Int J Mol Sci. 2023 Oct 25;24(21):15577. doi: 10.3390/ijms242115577 (PMC10650821; doi:10.3390/ijms242115577)

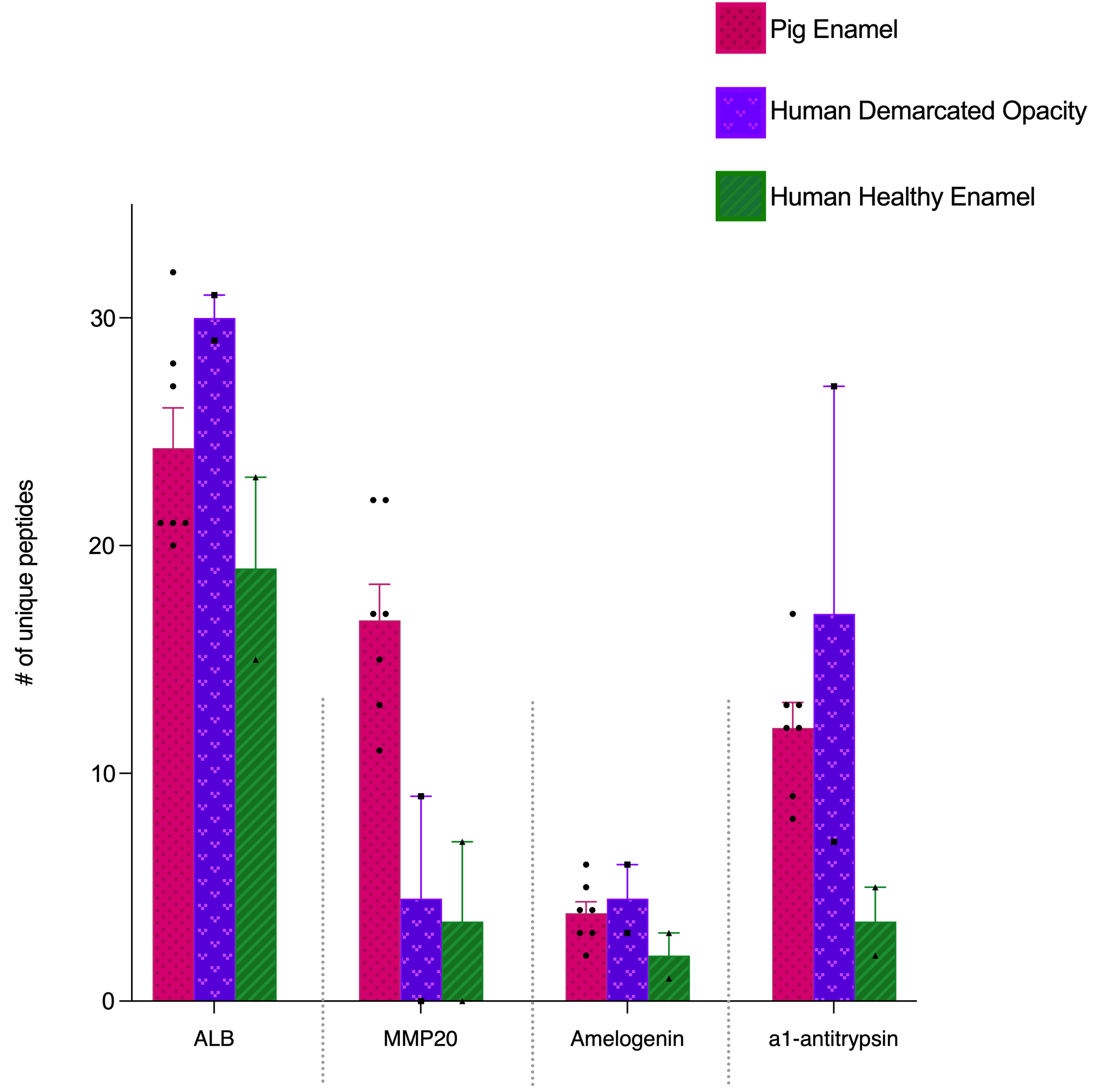

Supplement: Supplementary file 1 [file ijms-24-15577-s001.zip › Supplementary material/Supplementary_Figure_2.tiff]

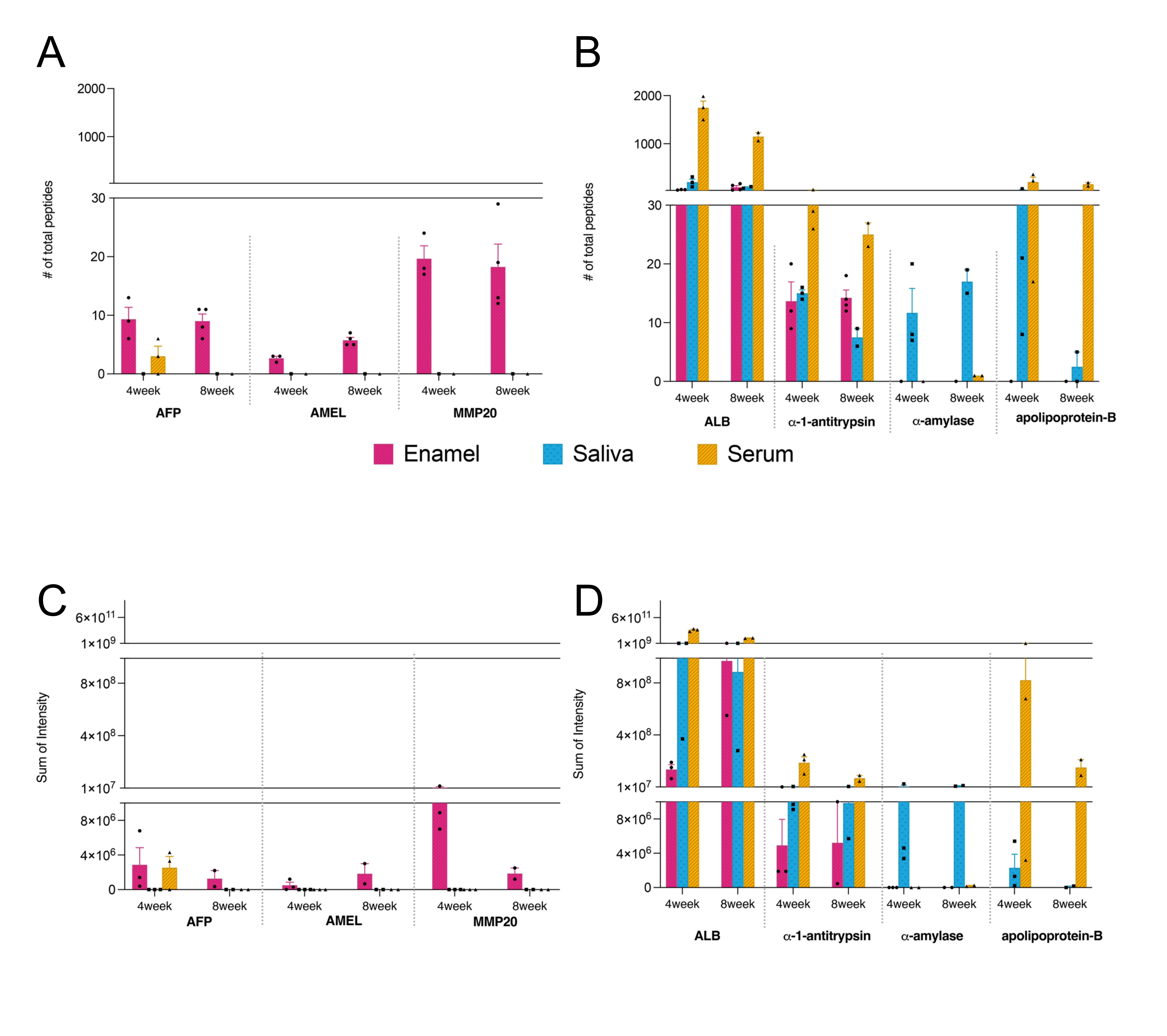

Supplement: Supplementary file 1 [file ijms-24-15577-s001.zip › Supplementary material/Supplementary_Figure_1.jpg]
